# Characterization of Novel Hepatitis B Virus PreS/S-Gene Mutations in a Patient with Occult Hepatitis B Virus Infection

**DOI:** 10.1371/journal.pone.0155654

**Published:** 2016-05-16

**Authors:** Jianhong Chen, Yan Liu, Jun Zhao, Zhihui Xu, Rongjuan Chen, Lanlan Si, Shanshan Lu, Xiaodong Li, Shuai Wang, Kai Zhang, Jin Li, Juqiang Han, Dongping Xu

**Affiliations:** 1 Institute of Infectious Diseases/Research Center for Clinical and Translational Medicine, Beijing 302 Hospital, Beijing 100039, China; 2 Department of liver disease, General Hospital of Beijing Military Region, Beijing 100700, China; University of Cincinnati College of Medicine, UNITED STATES

## Abstract

**Objective:**

The impact of hepatitis B virus (HBV) preS/S-gene mutations on occult HBV infection (OBI) is not fully understood. This study characterized multiple novel HBV preS/S-gene mutants obtained from an OBI patient.

**Methods:**

PreS/S-gene mutants were analyzed by clonal sequencing. Viral replication and expression were analyzed by transfecting HBV genomic recombinants into HepG2 cells.

**Results:**

Twenty-one preS/S-gene mutants were cloned from four sequential serum samples, including 13 mutants that were not previously documented: (1) sI/T126V+sG145R; (2) preS1 nt 3014−3198 deletion; (3) preS1 nt 3046−3177 deletion; (4) preS1 nt 3046−3177 deletion+s115−116 “INGTST” insertion; (5) preS1 nt 3046−3177 deletion+s115−116 “INGTST” insertion+sG145R; (6) preS1 nt 3115−3123 deletion+sQ129N; (7) preS1 nt 3115−3123 deletion+s126−127 “RPCMNCTI” insertion; (8) s115−116 “INGTST” insertion; (9) s115−116 “INGTST” insertion+sG145R; (10) s126−127 “RPCMNCTI” insertion; (11) preS1 nt 2848−2862 deletion+preS2 initiation codon M→I; (12) s122−123 “KSTGLCK” insertion+sQ129N; and (13) preS2 initiation codon M→I+s131−133TSM→NST. The proportion of preS1 nt 3046−3177 deletion and preS2 initiation codon M→I+s131−133TSM→NST mutants increased in the viral pool with prolonged disease. The 13 novel OBI-related mutants showed a 51.2−99.9% decrease in HBsAg levels compared with that of the wild type. Additional N-glycosylation-associated mutations, sQ129N and s131−133TSM→NST, but not s126−127 “RPCMNCTI,” greatly attenuated anti-HBs binding to HBsAg. Compared with the wild type, replication and surface antigen promoter II activity of the preS1 nt 3046−3177 deletion mutant decreased by 43.3% and 97.0%, respectively.

**Conclusion:**

PreS/S-gene mutations may play coordinated roles in the presentation of OBI and might be associated with disease progression. This has implications for HBV diagnosis and vaccine improvement.

## Introduction

Loss of HBsAg and anti-HBs seroconversion are considered signs of hepatitis B virus (HBV) elimination. However, serum/intrahepatic HBV DNA can be found in some patients who are negative for serum HBsAg. This status is defined as occult HBV infection (OBI) [1,2]. Research on the following aspects of OBI is increasing: (1) transmission through transfusion, parturition, organ transplantation, or hemodialysis; (2) reactivation during a state of immunosuppression; (3) contribution to the progression of chronic liver disease; and (4) increased risk for hepatocellular carcinoma [3–5].

HBV preS/S-gene mutation is one of the major causative factors for OBI. HBV envelope protein is encoded by the preS/S gene, which includes the preS1, preS2 and S genes. Promoter SPI [nucleotide (nt) 2219−2780] regulates the transcription of a 2.4-kb mRNA and encodes the large (L) protein. Promoter SPII (nt 2809−3152) regulates the transcription of a 2.1-kb mRNA and encodes the middle (M) and small (S) proteins. The main protein includes glycosylated GP27 and non-glycosylated P24. The region of amino acids (aa) 99−169 is termed major hydrophilic region (MHR), and it contains the major conformational epitope exposed on the external surface of the viral particle [6]. MHR N-glycosylation mutations may influence viral characteristics [7]. There is a relatively conserved region (aa 124−147) within the MHR called the “a” determinant, which is the target of neutralizing B cell responses [8,9].

The number of reported OBI varies greatly by population and region. One investigation showed that the prevalence of OBI reached 73% (24/33) in cryptogenic hepatocellular carcinoma (HCC) patients [10]. A population-based study revealed that the OBI prevalence in Chinese blood donors was 0.16% (61/38,499), and 14 different non-synonymous mutations in the MHR of the S gene were detected in 34 of these OBI blood donors. In this study, four mutations (sC124R, sC124Y, sK141E, and sD144A) strongly decreased the sensitivity of HBV detection in seven commercial HBsAg immunoassays [11]. Cheung et al. [12] reported on a patient with persistent OBI and lymphoma who harbored 6 non-synonymous mutations in the “a” determinant of the S gene. Recently, a novel vaccine escape S gene mutant (sP120Q+sD144A) was described. This mutant virus was transmitted through parturition to a vaccine-protected child and persistently replicated in the child for 3 years with undetectable HBsAg [4]. OBI-related preS/S-gene mutations documented in previous studies are summarized in Table 1 [4, 11–41]. In this study, we aimed to clarify the clinical and virological characteristics of multiple novel OBI-related preS/S-gene mutants derived from a unique OBI patient.

**Table 1:** Previously reported preS/S-gene mutations associated with OBI.

S gene point mutations in the MHR	sD99N, sY100C/F/S, sQ101K/R, sM103I, sL109P, sL110I, sP111L, sG112R, sT113N/S, sS114T/N, sT115A/N, sS117G/T, sG119R, sP120Q/T, sC121R, sK122R/N/I, sT123A/N/V, sC/T124S/R/Y, sT125A/M, sI/T126A/N/S, sP127T, sA128T, sQ129H/K/N/R, sG130N/R/S, sT131I/N/P, sS132P, sM133T, sF134L/V/Y, sS136P, sC137R/Y, sC138Y, C139R/Y, sT140I, sK141E, sP142L/S, sS143L/M, sD144A, sG145A/R, sN146S, sC147R, sC149R, sP151L, sF158L, sA159G, sK/R160N, sS167L
S gene point mutations outside of the MHR	sF19V, sS34L, sN40S, sA/T45K, sC48R, sL/P49I, sS55P, sP62L, sP70L, sY72H, sF80S, sI82M/T/V, sL84F, sF85C, sI86V, sL87Q, sF93L, sL95W, sV96I, sM197T, sS171F, sL173P, sS174G, sL176P, sW182L, sV190I, sW191R, sM198V, sS204R, sL205V, sS210G, sI218M, sF219L/S
Other S gene mutations	Premature stop mutations, MHR insertions
preS gene mutations	Deletions, premature stop mutations and point mutations in the preS1 gene; preS2 initiation codon substitution, point mutations, and premature stop mutations in the preS2 gene

## Methods

### Patient and materials

**Fig 1:**
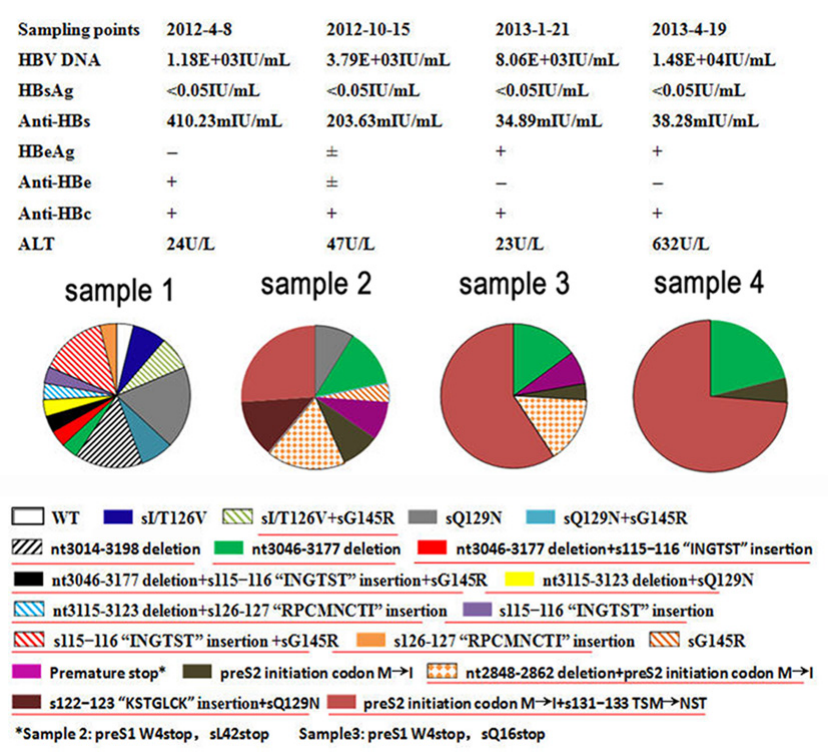
Dynamics of preS/S-gene mutant strains in four sequential samples. WT, wild-type; ±, weak positive. Novel OBI-related mutatants are underlined.

A patient was identified as positive for HBsAg in May 2004, with a serum HBV DNA level of 2.0 × 10^8^ copies/mL. The patient did not receive any treatment as an asymptomatic HBV carrier at that time. In January 2012, at the age of 63, the patient was hospitalized for the first time with a persistent low-grade fever and anorexia, and was recruited into the study. Based on serial examinations, the patient was diagnosed with advanced HBV-related HCC with intraperitoneal and pulmonary metastasis. The patient had an undetectable level of serum HBsAg, with 5.23 × 10^4^ copies/mL of serum HBV DNA. Adefovir (10 mg/d) was administrated to treat the HBV infection, but the virological response was poor. The patient died following multiple (including liver) organ failure in May 2014. Four sequential serum samples were collected from the patient and were stored at −40°C. The sampling time points and serological HBV markers for the four samples are shown in Fig 1. Written informed consent for the study was obtained from the patient. Ethical approval was given by the Ethics Committee of Beijing 302 Hospital.

### Polymerase chain reaction (PCR) amplification and sequencing of the HBV preS/S gene

HBV DNA was extracted from sera and the viral preS/S gene was amplified by nested PCR, using PSup3 and SB1R as upstream and downstream primers for the first round reaction, and PSup4 and SB2R as upstream and downstream primers for the second round reaction (Table 2). The PCR products were purified and cloned into pGEM-Teasy vectors (Promega, Madison, WI, USA) and then transformed into JM109 competent cells (Promega) for clonal sequence analysis. At least 20 clones of the viral preS/S gene were analyzed from each serum sample.

**Table 2:** PCR amplification primers.

Primers	Sequence of primers(5′→3′)	Binding sites	Application
PSup3	TCGCAGAAGATCTCAATCTCG	nt 2416−2436	preS/S gene amplification
SB1R	AGGTGAAGCGAAGTGCACAC	nt 1577−1596	preS/S gene amplification
PSup4	CATA AGGTGGGAAACTTTAC	nt 2466−2485	preS/S gene amplification
SB2R	TTCCGCAGTATGGATCGGCAG	nt 1258−1278	preS/S gene amplification
HISup	TCAGAATTCTCGAGGACTGGGGACCCTG	nt 125−146	S gene amplification
HISdown	AGCGGTACCAATGTATACCCAAAGACAA	nt 814−832	S gene amplification
P1up	CGGGGTACCTTTGTGGGTCACCATATTCTTG	nt 2809−2830	P1 /P3segment amplification
P1down	CTGATTTGCCTCTGGCCAATGA	nt 2997−3018	P1 segment amplification
P2up	CAATCCAGATTGGGACTTCAAC	nt 2964−2985	P2 segment amplification
P2down	GGAAGATCTGTTGTGGAGTTCCACTGCATGG	nt 3203−3225	P2 segment amplification
P3down	CAACTGGTGATCGGGAAAGAATC	nt 2915−2938	P3 segment amplification
P4up	CGACAAGGCATGGGGACGAATC	nt 2872−2894	P4 segment amplification
P4down	GGAAGA TCTCCTGACTGCCGATTGGTGGAGG	nt 3130−3152	P4 segment amplification

Sequencing was performed by Tian Yi Hui Yuan Biotech (Beijing, China) using an ABI 3730x1 DNA Analyzer (Applier Biosystems, Foster City, CA, USA). The deletion and insertion in the preS/S gene and point mutations associated with immune escape were analyzed using Vector NTI Suite 9.0 (Informax, Frederick, MD, USA). “Novel mutants” refers to virus clones harboring OBI-related preS/S-gene mutations (Table 1) and/or mutational patterns that have not been reported previously. The wild-type virus from the patient was a cloned sequence without an OBI-related mutation/mutational pattern. A phylogenetic analysis was performed with MEGA 4 software to genotype and evaluate the evolutionary relationship between cloned preS/S genes as we described previously [42, 43]. Multiple sequences were aligned using Lasergene MegAlign software (DNASTAR, Madison, WI, USA).

### Construction of recombinant vectors

**Fig 2:**
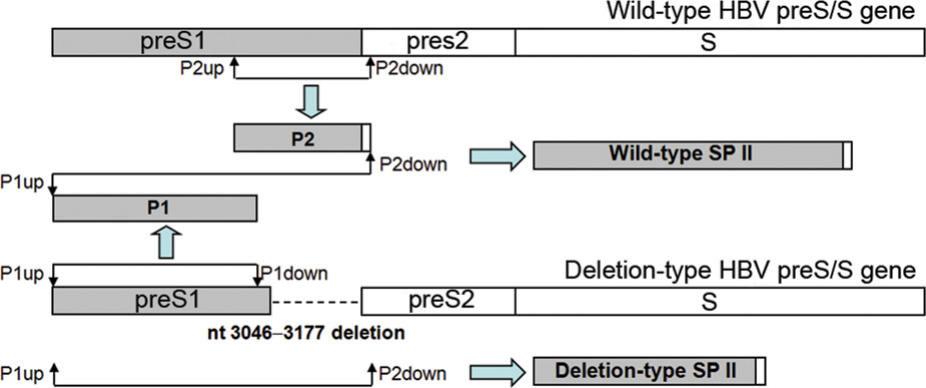
Diagram for obtaining deletion-type and wild-type SPII.

The following three sets of recombinant vectors were constructed: (1) The recombinant pTriEx-mod-1.1 HBV vector, which was a gift from Professor Zoulim, University Lyon, France. The recombinant vectors were constructed as we described previously [44], with minor modifications. Briefly, the pGEM-Teasy vector containing the wild-type or mutant preS/S gene was extracted and digested with *Bst*EⅡ and *Sph*Ⅰ. The target preS/S gene was inserted into the pTriEx-mod-1.1 HBV vectors digested with the same enzymes. (2) The pcDNA3.1(-)/myc-His vector, which was purchased from Invitrogen (Carlsbad, CA, USA). *Eco*RⅠ and *Kpn*Ⅰ enzyme sites were added to the target S region (nt 155−835) by PCR using HISup and HISdown primers (Table 2). The wild-type or mutant target S region was digested with *Eco*RⅠ and *Kpn*Ⅰ and linked to the pcDNA3.1(-)/myc-His A vector treated with the same enzymes. (3) HBV pGL3-SPII luciferase expression vector. Because the preS1 deletion (nt 3046−3177) mutant contained defective SPII (nt 2809−3152), both deletion-type and wild-type SPII recombinant pGL3-basic vectors were constructed to study the effect of the mutant on SPII activity. The protocol was designed to ensure that wild-type SPII and deletion-type SPII had the same background sequence. The protocol for obtaining the wild-type counterpart of the nt 3046−3177-deletion type preS1 gene is shown in a diagram in Fig 2. The primers P1up and P1down were used to amplify segment P1 using the deletion-type preS/S gene as a template. P2up and P2down were used to amplify segment P2 with wild-type preS/S gene as template. An overlapping PCR was performed with P1up and P2down to amplify wild-type SPII using P1 and P2 as templates. The deletion-type SPII was directly amplified with P1up and P2down using the deletion-type preS/S gene as a template.

The same strategy was used for construct the wild-type counterparts of two other deletion-type (nt 3014−3198 and nt 3115−3123) preS1 genes. To obtain the wild-type counterpart of the nt 2848−2862 deletion-type preS1 gene, P1up and P3down were used to amplify segment P3 with the deletion-type preS/S gene as a template. P4up and P4down were used to amplify segment P4 with wild-type preS/S gene as a template. After this, P3 and P4 were used as templates to amplify wild-type SPII using primers P1up and P4down. The segments containing deletion-type SPII were amplified using primers P1up + P2down or P1up + P4down with individual deletion-type preS/S genes as the templates. The target segments were digested with *Kpn*Ⅰand *Bgl*Ⅱ and inserted into pGL3-basic luciferase expression vectors. All constructs were confirmed by sequencing. The primers used are listed in Table 2.

### Quantitation of intracellular replicative intermediates, supernatant HBV DNA, and HBsAg

HepG2 cells were cultured in Dulbecco’s modified Eagle’s medium (DMEM, Gibco) containing 10% fetal bovine serum in optimal conditions. The cells were seeded into six-well plates (4 × 10^5^ cells/well). The recombinant pTriEx-mod-1.1HBV vectors were transfected into HepG2 cells mediated by X-treme GENE HD transfection reagent (Roche, Mannheim, Germany). As a control, the β-galactosidase reporter plasmid was co-transfected to normalize transfection efficiency. The transfection efficiencies within an experiment and across independent experiments were comparable. After cultivation, cells were harvested and lysed. Intracellular replicative intermediates were quantitated as we previously described [45]. In brief, four days after cultivation, cells were harvested and lysed. HBV replicative intermediates and supernatant HBV DNA were quantitated using a real-time quantitative PCR kit (Fosun Pharmaceutical Co., Ltd., Shanghai). Supernatant HBsAg were quantitated using a Roche Cobas e601 electrochemistry luminescence immunity analyzer and an Elecsys for HBsAg quantitation (Roche Diagnostics, Mannheim, Germany). The experiment was repeated at least three times independently.

### Western blot analysis of HBsAg

pcDNA3.1(-)/myc-His A recombinant vectors were transfected into HepG2 cells. After 3 days of cultivation, protein was obtained from cell lysates. A Western blot analysis was performed as previously described [46]. In brief, mouse monoclonal anti-HBs (Santa Cruz Biotechnology Inc., Santa Cruz, CA 95060, USA) and anti-His tag (Kang Wei Biotechnology Inc., Beijing, China) antibodies were used as primary antibodies. Goat-anti-mouse monoclonal antibody was used as a secondary antibody. β-actin was used as an internal control and detected by anti-β-actin.

### Immunofluorescence analysis of His-tagged HBsAg

HepG2 cells grown on glass coverslips and transfected with pcDNA3.1(-)/myc-His-S plasmids were briefly washed with phosphate buffered saline (PBS) twice on day 3 post transfection, fixed using 4% paraformaldehyde, permeabilized with 0.1% Triton X-100, and blocked with 3% blocking buffer. The cells were incubated with horse anti-HBs (Abcam, Cambridge, United Kingdom) and mouse anti-His tag (Zhong Shan Jin Qiao Biotech Inc., Beijing, China), followed by an incubation with Cy3-conjugated rabbit-anti-horse IgM/Alexa Fluor 488 (Bo Ao Sen Biotech Inc., Beijing, China) and goat-anti-mouse IgG (Kang Wei Shi Ji Biotech Inc., Beijing, China) antibodies. The cells were examined by laser scanning confocal microscopy. The fluorescence intensity was analyzed using Image-Pro Plus software (Media cybernetics Inc., Rockville, USA).

### Dual-luciferase reporter gene assay

HepG2 cells were seeded into 24-well plates (3 × 10^5^ cells/well). HBV pGL3-SPII luciferase expression vectors and internal control pRL-TK vectors (0.3 μg/well *vs*. 0.01 μg/well) were co-transfected into HepG2 cells. pGL3-Basic and pGL3-Control were used as negative and positive controls, respectively. After 48 h of cultivation, cells were lysed. Luciferase activity was detected and corrected by the internal control using a dual-luciferase reporter gene assay kit (Promega) and Synery H4 Hybrid Reader (Bio Tek Instruments, Inc., Winooski, VT, USA). The transfection experiment was repeated 3 times, with 2 independent culture dishes each time.

### Statistical analysis

Data are presented as the mean ± standard deviation. Differences between variables were examined by Student’s *t*-test. The statistical analysis was carried out in Statistical Program for Social Sciences (SPSS 18.0 for Windows; SPSS Inc., Chicago, IL, USA). A *P*-value of < 0.05 (2-tailed) was considered statistically significant.

## Results

### HBV sequencing and phylogenetic analysis

All cloned viral sequences from the patient were belonged to the HBV/C genotype. Phylogenic analysis showed that the cloned sequences were closest evolutionarily to sequence Y18856 deposited in GenBank. Compared to the Y18856 sequence, 21 classical and unusual preS/S-gene mutants that may be related to OBI were detected in the viral pool (Fig 1). The mutational patterns included point mutations in the “a” determinant region, premature stop mutations, preS1 large fragment deletions, a preS2 initiation codon mutation, MHR region insertions, an N-glycosylation-introduced mutation, and a classical immune-escape mutation. These mutational patterns existed both singly or in combination in the viral gene. The multiple sequence alignment is shown in S1 Fig.

Thirteen novel OBI-related mutants were detected. These mutation were the following: (1) sI/T126V+sG145R (KR014124); (2) preS1 nt 3014−3198 deletion (KR014127); (3) preS1 nt 3046−3177 deletion (KR014128); (4) preS1 nt 3046−3177 deletion+s115−116 “INGTST” insertion (KR014129); (5) preS1 nt 3046−3177 deletion+s115−116 “INGTST” insertion+sG145R (KR014130); (6) preS1 nt 3115−3123 deletion+sQ129N (KR014131); (7) preS1 nt 3115−3123 deletion+s126−127 “RPCMNCTI” insertion (KR014132); (8) s115−116 “INGTST” insertion (KR014133); (9) s115−116 “INGTST” insertion+sG145R (KR014134); (10) s126−127 “RPCMNCTI” insertion (KR014135); (11) preS1 nt 2848−2862 deletion+preS2 initiation codon M→I (KR014141); (12) s122−123 “KSTGLCK” insertion+sQ129N (KR014142); and (13) preS2 initiation codon M→I+s131−133TSM→NST (KR014143).

The proportions of the mutant and wild-type strains in the viral pools from 4 sequential serum samples are shown in Fig 1. The mutant with a preS1 large fragment deletion (nt 3046−3177 deletion) was detected in 3.7%, 13.0%, 14.8%, and 21.1% of detected virus clones, and the mutant with preS2 initiation codon M→I+s131−133TSM→NST was detected in 0%, 26.1%, 59.3%, and 73.7% of detected virus clones, respectively. Drug-resistance testing was performed after adefovir treatment, and no resistance mutations were detected.

### Quantitation of intracellular replicative intermediates, supernatant HBV DNA, and HBsAg

**Fig 3:**
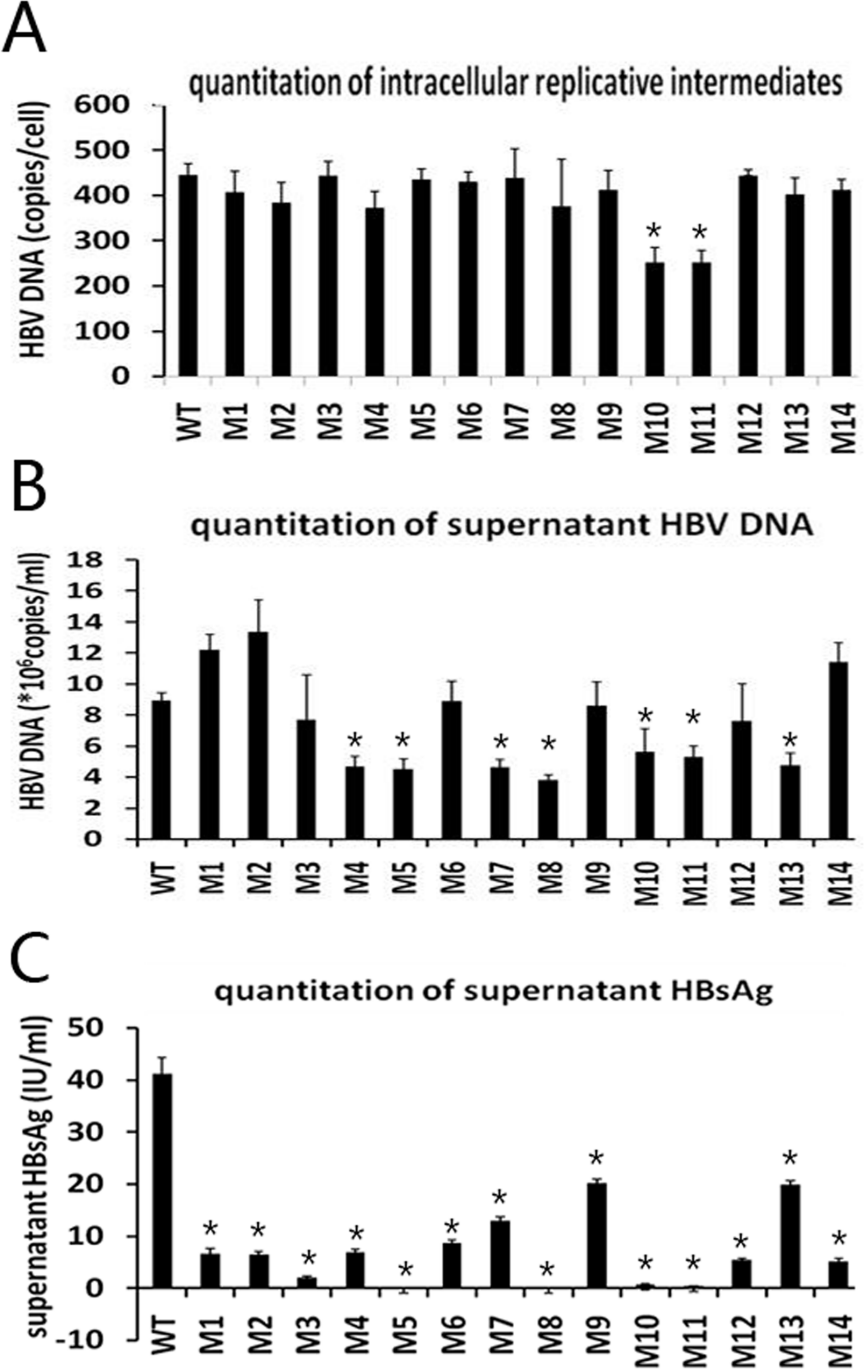
**Quantitation of intracellular replicative intermediates (A), supernatant HBV DNA (B), and supernatant HBsAg (C).** WT, wild-type; M1, sQ129N; M2, s131−133TSM→NST; M3, sI126V; M4, sG145R; M5, sI126V+sG145R; M6, s115−116 “INGTST” insertion; M7, s115−116 “INGTST” insertion+sG145R; M8, s122−123 “KSTGLCK” insertion+sQ129N; M9, s126−127 “RPCMNCTI” insertion; M10, nt 3014−3198 deletion; M11, nt 3046−3177 deletion; M12, preS2 initiation codon M→I+s131+133TSM→NST; M13, nt 2848−2862 deletion+preS2 initiation codon M→I; M14, nt 3115−3123 deletion+sQ129N (* *P* < 0.05, mutant *vs*. WT).

A phenotypic analysis was performed for 14 mutants (M1−M14), which were either dominant/subdominant in the viral pool or emerged serially, as well as for one wild-type strain. Compared with the wild-type strain, intracellular replicative intermediate level of M10 and M11 decreased by 43.5% and 43.3%, respectively. The other mutants did not show a significant change (Fig 3A). Supernatant HBV DNA levels of M7, M8, M10, M11, and M13 decreased by 48.0%, 57.2%, 38.2%, 40.8%, and 46.6%, respectively; while M1 and M2 increased by 36.5% and 49.6%, respectively (Fig 3B). Supernatant HBsAg levels were all significantly decreased. Specifically, M1−M13 decreased by 84.1%, 84.6%, 95.4%, 83.4%, 99.9%, 80.0%, 68.8%, 99.9%, 51.2%, 98.3%, 99.1%, 87.0%, and 52.0%, respectively (Fig 3C).

### Western blot analysis of His-tagged HBsAg

**Fig 4:**
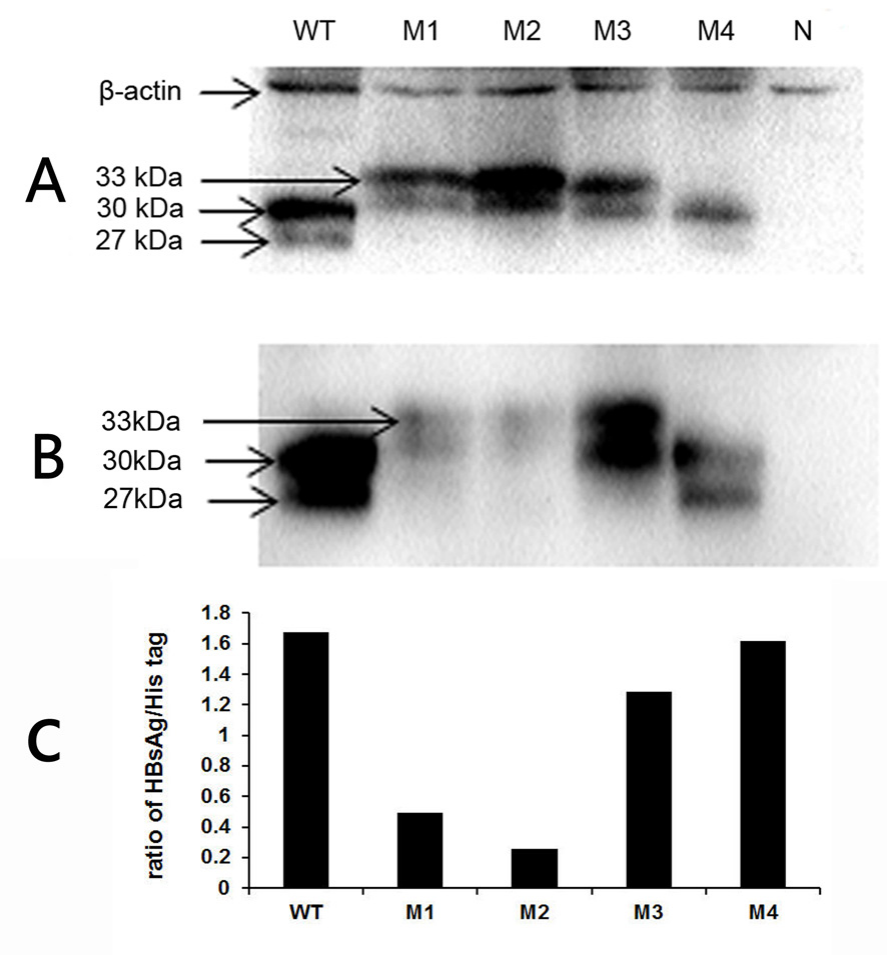
Western blot analysis of His-tagged HBsAg. His-tagged target proteins were detected using anti-His tag monoclonal antibody (A) or anti-HB monoclonal antibody (B). A relative densitometry analysis of the bands was performed using a Tanon gel image system (C). WT, wild-type; M1, sQ129N; M2, s131−133TSM→NST; M3, s126−127 “RPCMNCTI” insertion; M4, sG145R; N, negative control.

Three mutants (M1, M2, and M3) were selected for western blot analysis, because they had N-glycosylated mutations (NXT) in the “a” determinant. The viral strain M4, harboring no additional glycosylation-associated mutations, was used as a reference. All His-tagged target proteins were detected, indicating that all fused genes were successfully expressed (Fig 4A). M1 and M2 mutants with N-glycosylation-associated mutations in the MHR showed significantly reduced binding (by 70.3% and 84.5%) of anti-HBs to target HBsAg compared to that of the wild-type mutant. By contrast, M3 with the s126−127 “RPCMNCTI” insertion, which also introduced an additional N-glycosylated site, showed reduced binding (by 23.2%) of anti-HBs to target HBsAg; M4 (sG145R) had little influence on this binding (Fig 4B and 4C). The M1, M2, and M3 mutants ran slower in polyacrylamide gel electrophoresis compared with the wild-type strain, possibly because each of the three mutations introduced an additional N-glycosylated site.

### Immunofluorescence analysis of His-tagged HBsAg

The binding of anti-HBs to target HBsAg was reduced by 61.2%, 91.6%, and 34.7% for M1, M2, and M3, respectively, compared to that of the wild-type strain. M4 showed no change in the binding of anti-HBs to target HBsAg (S2 Fig).

### Relative luciferase activity analysis

A dual luciferase reporter gene assay showed that the relative luciferase activity (firefly luciferase expression of a deletion-type or wild-type strain / Renilla luciferase expression of an internal control) of the wild-type strain was 42.15 ± 0.86. Compared with the wild-type strain, the relative luciferase activity of the mutants with deletions in nt 3014−3198, nt 3046−3177, and nt 2848−2862 were 1.94 ± 0.13, 1.26 ± 0.07, and 26.97 ± 1.35, showing decreases of 95.4%, 97.0%, and 36.0%, respectively (*P* < 0.05). The deletion at nt 3115−3123 showed no significant effect on the relative luciferase activity.

## Discussion

In this study, we identified a unique OBI patient who harbored 21 HBV preS/S-gene mutants, including 13 novel OBI-related mutants. As the basis of this finding, we investigated over 30,000 patients and blood donors, and sequenced HBV genes of 71 OBI subjects’ samples in the investigation population. HBV preS and/or S gene mutations, summarized in Table 1, were detected in 35 OBI subjects. The 13 novel OBI-related preS/S-gene mutations were only detected in this patient, although the sQ129N, s131−133TSM→NST, and sG145R mutations were detected in one, three, and one, respectively, of the other 34 subjects. The patient studied here was the most noteworthy subject because the HBV preS/S-gene mutation profile in this subject was the most complex, with dynamic changes in his four sequential serum samples. In addition, we analyzed 516 HBV full-length genomic sequences and 18,419 HBV RT/S-gene sequences from individual patients that were described in our previous studies [44, 47–49]. We also analyzed 13,138 preS/S region sequences obtained from GenBank, including 7,099 complete genomic sequences. None of the 13 novel OBI-related mutations/mutational patterns was detected in these other sequences.

We performed a dual luciferase reporter gene assay to determine whether a preS deletion could alter SPII promoter activity. Results showed that the relative luciferase activity of preS1 large fragment deletion strains (nt 3046−3177 and nt 3014−3198) decreased by over 95% compared with that of the wild-type strain, indicating that preS1 large fragment deletions dramatically decrease SPII promoter activity. This may lead to the large decrease in 2.1-kb mRNA transcription and consequent low HBsAg expression, as shown in Fig 3C. A short fragment deletion in the region had less or very little influence.

sG145R has been reported as a classical immune escape mutation in the “a” determinant. Regarding the mechanism, some studies have suggested that the sG145R mutation reduces the antigenicity of HBsAg [18,50], while other studies have suggested that this mutation impairs the capacity for HBsAg or viral particles to be secreted [51–53]. Our results showed that the sG145R mutation had little effect on the binding of anti-HBs to HBsAg (Fig 4) or intracellular replicative intermediate levels (Fig 3A), but significantly decreased supernatant HBV DNA levels (Fig 3B). In this study, sG145R was mainly detected in combination with other mutations in the MHR, including sI/T126V, sQ129N, and the s115−116 “INGTST” insertion. The combined mutations may endow an even greater antigen reduction.

Additional N-glycosylation-associated mutations in the MHR have been reported to attenuate HBsAg antigenicity and to contribute to immune escape [7]. Our study verified that sQ129N and s131−133TSM→NST significantly attenuated binding of anti-HBs to HBsAg, but that the s126−127 “RPCMNCTI” insertion, which also introduced an additional N-glycosylation site, had much weaker influence. Interestingly, the decrease in detectable virus from samples 1 to 3 was accompanied by a decline in anti-HB levels. Considering that the strain with the s126−127 “RPCMNCTI” insertion showed a nearly normal ability to bind to anti-HBs and was only detected in sample 1, we speculate that this novel mutant (possibly along with some other mutants that subsequently disappeared) might elicit anti-HBs that interfere with HBsAg detection.

Different mechanisms of mutation in HBV may account for the presentation of OBI in this patient: (1) the preS1 large fragment deletion reduced viral replication and expression; (2) the S gene mutations reduced HBsAg antigenicity; (3) mutant HBsAg might have elicited low-affinity or non-neutralizing antibodies that interfered with detection by the HBsAg reagent, as the anti-HBs in all 4 serum samples were positive. Thus, multiple preS/S gene mutations may play a coordinated role in determining the presentation of OBI in this patient.

We found that the proportion of the mutant strains with the preS1 large fragment (nt 3046−3177) deletion and the mutant strains with the preS2 initiation codon M→I+s131−133 TSM→NST increased across four sequential samples, suggesting that the two viral strains displayed greater replication competence than other viral strains. On one hand, the mutant strains may have escaped the immune response, which may partly explain the association of HBV S gene mutations with disease progression. In addition, the preS1 large fragment deletion may have an oncogenic effect. Some studies have demonstrated that viral strains with the preS1 large fragment deletion can produce a truncated large protein that may accumulate in the endoplasmic reticulum and cause endoplasmic reticulum stress and oxidative DNA damage, and accelerate the progression of liver disease and tumorigenesis [5, 54–56]. Notably, the patient received adefovir not long before the first sample was taken. Although the drug showed no effect on the total viral load, it might have selectively suppressed some viral strains. Thus, antiviral-virus and host-virus interactions may play coordinated roles in the evolution of HBV in this case.

Only four sequential serum samples were available from this patient. This limited our ability to associate viral mutations with disease progression. Nevertheless, some interesting issues arise from this case, such as the impact of individual novel mutations in HBsAg on diagnostic assays, complementary interactions among individual mutants, and the potential for horizontal transmission of viral mutants in vaccinated people. These issues require further study.

The major novelties of this study are the discovery of 13 novel preS/S-gene mutants, clarification of the evolution of the novel mutants through the course of disease, and phenotypic characterization of the major novel mutants. Taken together, these results suggest that multiple preS/S-gene mutations may play coordinated roles leading to OBI and might be associated with disease progression. This study provides new insights into OBI that will be helpful in OBI diagnosis and management, as well as to improve HBV vaccines.

## Supporting Information

S1 Fig **Multiple sequence alignments of the HBV S region (A) and preS region (B).** Amino acid sequences of 21 mutants and one wild-type strain cloned from four sequential samples obtained from the patient and 50 reference sequences of HBV genotype C from NCBI were aligned using Lasergene MegAlign software. The sequences are labeled with their GenBank accession numbers. * represents reference sequences of genotype C.
(TIF)

S2 Fig Immunofluorescence analysis of His-tagged HBsAg. His-tagged HBsAg were detected using mouse anti-His tag monoclonal antibody and horse anti-HBs monoclonal antibody, followed by different fluorescence-conjugated secondary antibodies (A) Relative densitometry analysis of fluorescence intensity (B) WT, wild-type; M1, sQ129N; M2, s131−133TSM→NST; M3, s126−127 “RPCMNCTI” insertion; M4, sG145R.
(TIF)
