# Supplementary figures and images for: Characterization of Novel Hepatitis B Virus PreS/S-Gene Mutations in a Patient with Occult Hepatitis B Virus Infection
Source: PLoS One. 2016 May 16;11(5):e0155654. doi: 10.1371/journal.pone.0155654 (PMC4868315; doi:10.1371/journal.pone.0155654)

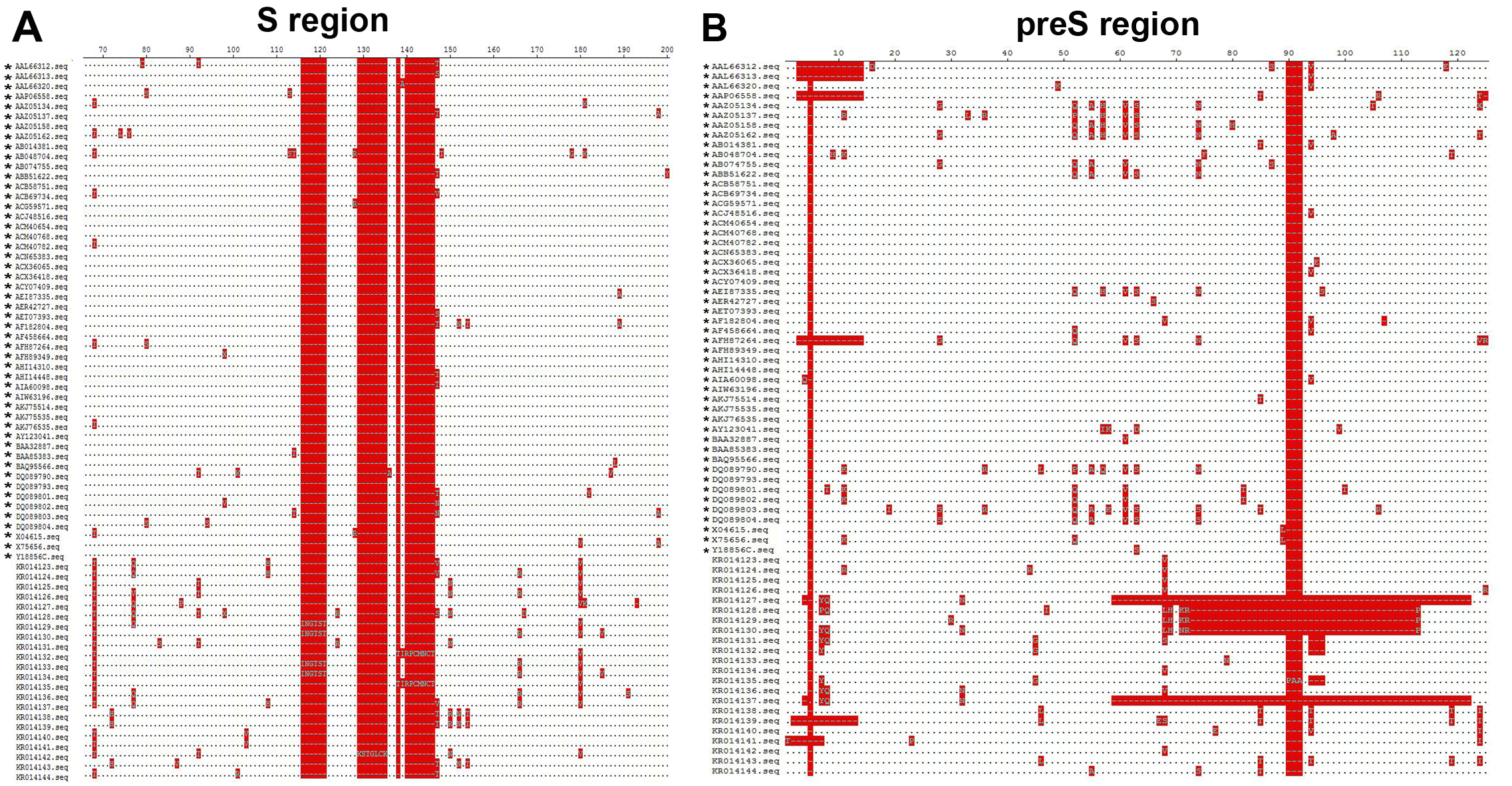

Supplement: S1 Fig — Multiple sequence alignments of the HBV S region (A) and preS region (B). Amino acid sequences of 21 mutants and one wild-type strain cloned from four sequential samples obtained from the patient and 50 reference sequences of HBV genotype C from NCBI were aligned using Lasergene MegAlign software. The sequences are labeled with their GenBank accession numbers. * represents reference sequences of genotype C. (TIF) [file pone.0155654.s001.tif]

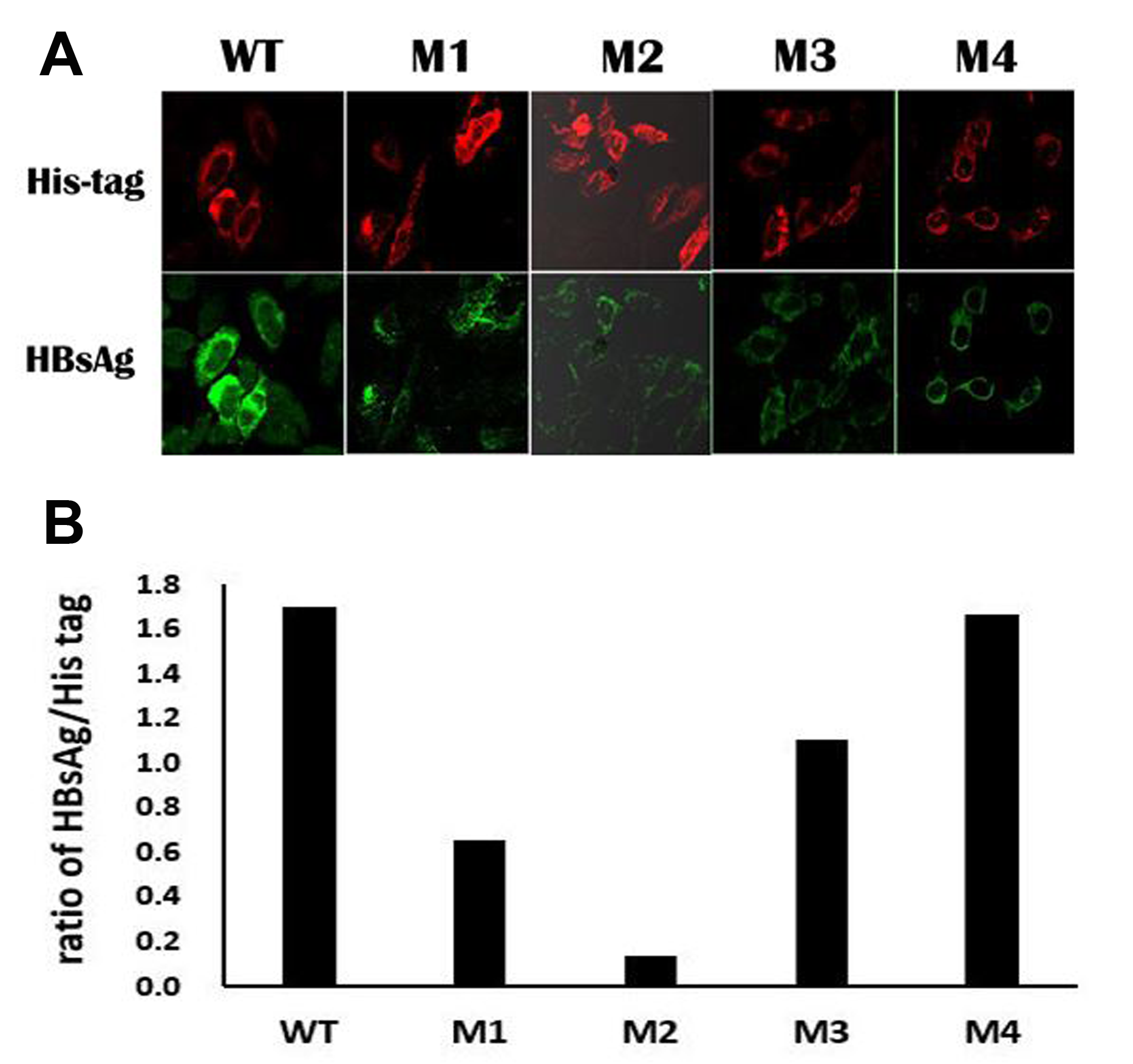

Supplement: S2 Fig — His-tagged HBsAg were detected using mouse anti-His tag monoclonal antibody and horse anti-HBs monoclonal antibody, followed by different fluorescence-conjugated secondary antibodies (A) Relative densitometry analysis of fluorescence intensity (B) WT, wild-type; M1, sQ129N; M2, s131−133TSM→NST; M3, s126−127 “RPCMNCTI” insertion; M4, sG145R. (TIF) [file pone.0155654.s002.tif]
